# Physico-Chemical Characterization and Initial Evaluation of Carboxymethyl Chitosan–Hyaluronan Hydrocolloid Systems with Insulin Intended for Intranasal Administration

**DOI:** 10.3390/ijms251910452

**Published:** 2024-09-27

**Authors:** Roxana Popescu, Cristina-Elena Dinu-Pîrvu, Mihaela Violeta Ghica, Valentina Anuța, Lăcrămioara Popa

**Affiliations:** 1 Department of Physical and Colloidal Chemistry, Faculty of Pharmacy, “Carol Davila” University of Medicine and Pharmacy, 6 Traian Vuia Str., 020950 Bucharest, Romania; roxana_popescu@drd.umfcd.ro (R.P.); cristina.dinu@umfcd.ro (C.-E.D.-P.); valentina.anuta@umfcd.ro (V.A.); lacramioara.popa@umfcd.ro (L.P.); 2 Innovative Therapeutic Structures Research and Development Centre (InnoTher), “Carol Davila” University of Medicine and Pharmacy, 020956 Bucharest, Romania

**Keywords:** carboxymethyl chitosan, intranasal insulin, nose-to-brain delivery

## Abstract

The nasal route of administration can bypass the blood–brain barrier in order to obtain a higher concentration in the brain, thus offering a feasible alternative route of administration for diseases associated with the central nervous system. The advantages of the intranasal administration and the potential favorable therapeutic effects of intranasally administered insulin led to the formulation of carboxymethyl chitosan (CMC) and sodium hyaluronate (NaHA) hydrocolloidal systems with insulin for nasal administration, targeting nose-to-brain delivery and the initial assessment of these systems. The influence of the formulation variables on the response parameters defined as surface properties, rheology, and in vitro release of insulin were analyzed using experimental design and statistical programs (Modde and Minitab software). The systems recorded good wetting and adhesion capacity, allowing the spread of the hydrocolloidal systems on the nasal mucosa. The samples had a pseudoplastic flow and the rapid release of the insulin was according to our objective. According to the physico-chemical characterization and preliminary assessment, these formulations are appropriate for administration on the nasal mucosa, but further studies are necessary to demonstrate the beneficial therapeutic actions and the safety of using intranasal insulin.

## 1. Introduction

Among researchers, more interest is shown toward the intranasal route of administration from the point of view of the management of neurodegenerative diseases and brain disorders (brain tumors, cerebral ischemic injuries, stroke and others), and for the administration of substances that act at the brain level, because it avoids the blood–encephalic barrier (BBB), enzymatic degradation, hepatic metabolism, and gastrointestinal pH issues [1,2,3,4]. Nasal administration is not invasive and does not require the presence of qualified personnel, thus improving the patient’s compliance with the treatment [1,5,6]. The nose-to-brain delivery of the active pharmaceutical ingredient is mostly achieved through the olfactory and trigeminal nerves, and also by systemic circulation, because the nasal mucosa is well vascularized [5,7,8,9].

For more over 100 years, insulin has been widely used for its hypoglycemic effect to treat diabetes [10,11], and lately the intranasal route has been studied for the administration of insulin [12]. People with Type 2 Diabetes Mellitus (T2DM) are more vulnerable to developing cognitive impairment [13,14], Alzheimer disease [15,16], or Parkinson disease [17,18].

To date, based on the information available on the clinicaltrials.gov website, there are studies that investigate the effectiveness of intranasal insulin administration for metabolic-, neurodegenerative- and central nervous system-associated diseases [19].

Currently, insulin is also being researched for its neuroprotective effect after intranasal administration, in addition to its use in the treatment of diabetes. Studies conducted have revealed the potential beneficial therapeutic action of insulin administered intranasal, by slowing the progression of neurodegenerative diseases, improving the cognitive impairments associated with T2DM, or stimulating the socio-communicative capacity of patients [20,21,22] (Alzheimer disease [23,24,25], Parkinson disease [26,27,28], eating disorders [29,30], but additional studies are still required.

It was shown that insulin can improve cognitive function by reducing amyloid plaques, influences tau phosphorylation, stabilizes microtubules, and enhances tubulin polymerization [1,31].

Even if the risk of hypoglycemia is lower after the administration of insulin intransally, compared with subcutaneous administration [32], there were some reported side effects during the clinical trials, such as flu-like symptoms, falls, hypoglycemia, dizziness, diarrhea [33], a slow decrease in blood glucose, nasal burning sensation [34], local nasal rhinitis [35], transient nasal stinging, nasal irritation, and unpleasant odor [36].

It was also observed that insulin may have a favorable impact on the management of addictions [37] and smoking behavior [38]. Based on a randomized study, 60 IU insulin was administered nasally daily to people who wanted to quit smoking, which showed a decrease in the need for nicotine, although the results are not yet conclusive. Also, adverse events like nasal irritation, sweating, confusion, watering eyes, anxiety, and others were reported [39,40].

The administration of a high cumulative nasal dose does not lead to an increase in the systemic concentration of insulin. Most of the studies carried out so far used a daily dose of 0.5–1.5 IU/body kg (or 10–160 IU/day) [41]. Studies conducted on small groups of people did not lead to serious adverse events, but at a dose higher than 160 IU/day, side effects may occur [15]. Further investigation needs to be performed to establish the therapeutic effect of insulin after intranasal administrations and its safety profile.

Carboxymethyl chitosan (CMC) is a derivative of chitosan obtained by introducing the carboxymethyl group into its structure, which can increase the water solubility of chitosan [42]. It is a biodegradable, biocompatible, non-toxic polymer [43,44]. Chitosan is very well studied because it has favorable mucoadhesion on the mucosa [45,46], but the mucoadhesive property of CMC is superior to chitosan [47] and acts as a permeation-enhancer adjuvant [48]. CMC has intrinsic actions, such as antibacterial [49], antioxidant, antifungal, anticancer, and antitumor [43].

Sodium hyaluronate (NaHA) (also known as hyaluronan) is the sodium salt of hyaluronic acid, a natural anionic polysaccharide and soluble in water. This substance possesses mucoadhesive properties, increases the viscosity of formulations, prolongs the contact time, and potentiates the absorption of proteins through the nasal mucosa [50,51]. It has the capacity to hydrate and protects against irritation [52] and also lubricates tissues [51].

It is necessary to consider the tight junction of the epithelium, the mucociliary clearance, and the optimal nasal pH for the formulation of the intranasal delivery systems. The factors that influence the release of the ingredient at the level of the olfactory area in the nasal cavity are surface tension, mucoadhesion, and viscosity [1].

Inspired by the potential therapeutic benefits of insulin administered intranasal, the aim of this study was the development and physico-chemical characterization of nasal systems based on CMC and NaHA with insulin, which would combine the mucoadhesive properties of the polymers and stimulate insulin absorption. We assessed the influence of formulation factors on surface properties, rheology, and in vitro release of insulin complementary using Design of Experiments strategies.

## 2. Results

### 2.1. Physico-Chemical Characterization of Hydrocolloidal Systems

#### 2.1.1. Visual Aspect and pH

The hydrocolloidal systems were clear, transparent, and homogeneous, without any suspended particles or other instabilities. The pH of the formulations was between 6.57 and 6.72, as shown in Table 1, which is in the range of the nasal tolerability.

The pH values were similar, and we can conclude that the formulation parameters do not have an impact on the pH.

#### 2.1.2. Contact Angle

The wetting capacity of the hydrocolloidal systems was characterized based on the contact angle (CA) determination. The average CA values were obtained from the left and right angle of the drop, at the contact with the solid surface. All the systems had the CA under 90°. The lowest contact angle was obtained for S7, 50.47 ± 0.26°, and the highest value was for S3, 68.79 ± 1.56°.

For the systems with a higher amount of insulin and the same CMC concentration and CMC/NaHA ratio, the CA is lower, especially for samples S3 and S7, where there is the most significant difference (18.32°), followed by the S4–S8 difference (6.25°).

#### 2.1.3. Surface Tension

The values obtained for the ST parameter varied between 57.19 ± 0.24 mN/m for S1 and 62.46 ± 0.29 mN/m for S8. The concentration of the CMC had a slight influence on the ST parameter; the values were slightly lower for the systems with CMC 1% *w*/*v*, except for samples S5 and S6, where the S5 had the highest value, but the difference was non-significant (1.9 mN/m).

To have good system tolerability by the nasal mucosa, the surface tension of the products should be appropriate to the nasal mucosa superficial tension. Systems S1, S3, S6, and S7 demonstrated this requirement, but samples S2 and S5 were also close.

#### 2.1.4. Work of Adhesion, Work of Cohesion, and Spreading Coefficient Based on Superficial Properties

The three parameters should be analyzed together because they are interconnected and offer information regarding the display and the adherence of the system on the nasal mucosa, indicated by a *p* value of 0.014. A high value of work of adhesion (Wa) combined with a lower work of cohesion (Wc) provide an optimal spreading coefficient (S).

For all the systems, the work of adhesion calculated based on the CA and ST was over 80 mN/m. The maximum value was for S7 (97.62 ± 1.1 mN/m), followed by S8 (95.17 ± 0.45 mN/m). The lowest value was determined for S3 (80.14 ± 2.75 mN/m), followed by S1 (82.84 ± 0.27 mN/m).

The weakest cohesion forces were recorded for S1 (114.38 ± 0.48 mN/m) and the strongest bonding was for S4 (124.14 ± 1.43 mN/m) and S8 (124.93 ± 0.23 mN/m).

Based on the results from Table 2, it can be stated that the formulations that have a better displaying capacity are the ones that have a spreading coefficient closer to zero: S7 (−21.68 ± 0.23 mN/m), S8 (−29.76 ± 0.21 mN/m), and S1 (−31.54 ± 0.7 mN/m).

For the superficial parameters determined for the eight hydrocolloidal systems, we can emphasize that all systems have a good adhesion capacity and a satisfactory spreading capacity.

### 2.2. Rheology Analysis

The power law model is suitable to evaluate the polymeric material and was applied to analyze the rheological flow of the systems. This model describes the relationship between the shear stress, shear rate, and viscosity [53]. According to Table 3, the determination coefficient values were between 0.9926 and 0.9980. The power law model adequately and correctly fit the rheological analysis, since all the values were higher than 0.9900. The flow index values were less than 1, which means that all the systems had non-Newtonian pseudoplastic behavior.

The higher consistency index values were recorded for the hydrocolloid systems that had the higher CMC concentration for the same amount of insulin, and the same tendency was noticed at the same concentration of CMC and insulin with the CMC/NaHA ratio (S1 < S3 and S2 < S4 for the lower amount of insulin, respectively, S5 < S7 and S6 < S8).

**Figure 1:**
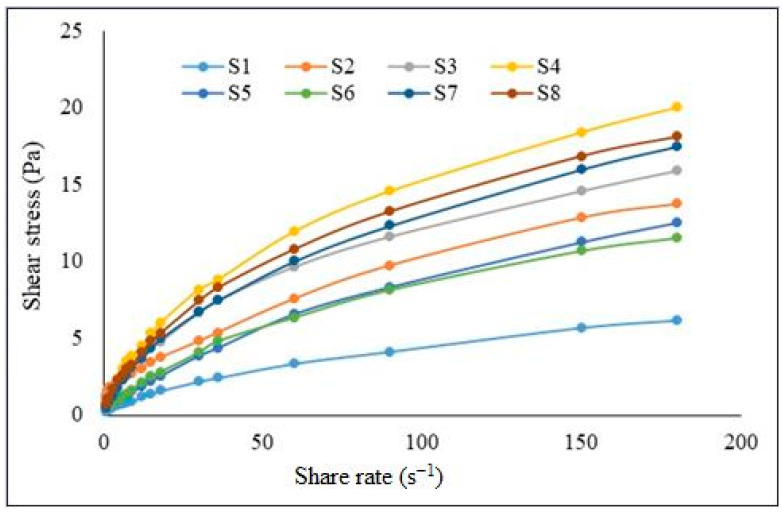
The flow curves representing the shear stress as function of shear rate.

The ascending flow curves of shear stress versus shear rate for all the systems are plotted in Figure 1, where the shear rate varies between 0.36 s^−1^ and 73.38 s^−1^.

**Figure 2:**
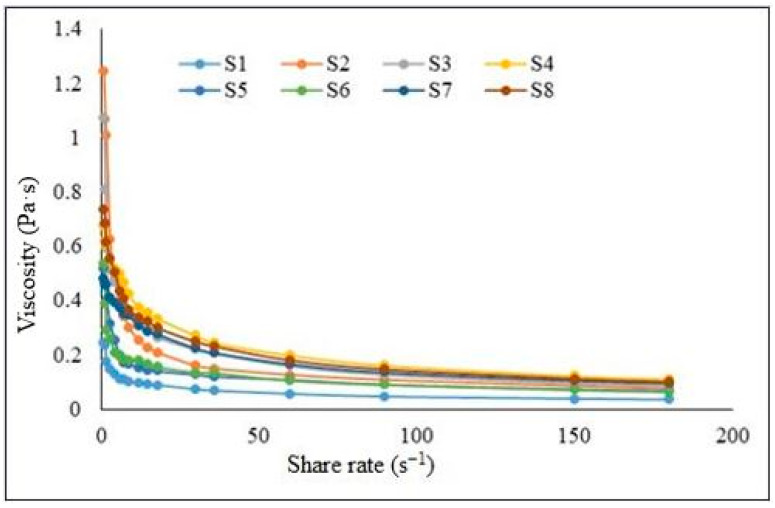
The influence of shear rate on the viscosity.

All the formulations presented shear-thinning characteristics; the viscosity decreased when the shear rate increased. The rheogram viscosity as function of shear rate is presented in Figure 2 for each hydrocolloidal system.

### 2.3. In Vitro Drug Release

The rapid release of the active pharmaceutical ingredient from the systems should be in accordance with the nasal characteristics. Different release kinetics models were applied (Higuchi, zero-order, and power law kinetic models), and the determination coefficients along with the kinetics parameters and the cumulative percentage of insulin release after 3 h are shown in Table 4. From the comparison of R^2^, the mathematical model that fit the experimental data best for the S1, S4, S7, and S8 formulations was the zero-order model, with a determination coefficient greater than 0.9934, and the values of the release rate of the insulin were between 0.342 min^−1^ and 0.538 min^−1^. The power law model fit the other systems better, where R^2^ was higher than 0.9958.

All the samples had over 60% diffusion through the membrane. System S1 released the highest amount of insulin, 95.91%, which was followed by S5 at 91.53%.

Based on these results, we can conclude that for the same amount of insulin, the highest cumulative drug release was for the systems with CMC 1% *w*/*v*. We can also compare for the same amount of insulin and the same concentration of CMC; for this situation, the samples with the CMC/NaHA ratio 1/1 had a better release versus the 1/2 ratio.

**Figure 3:**
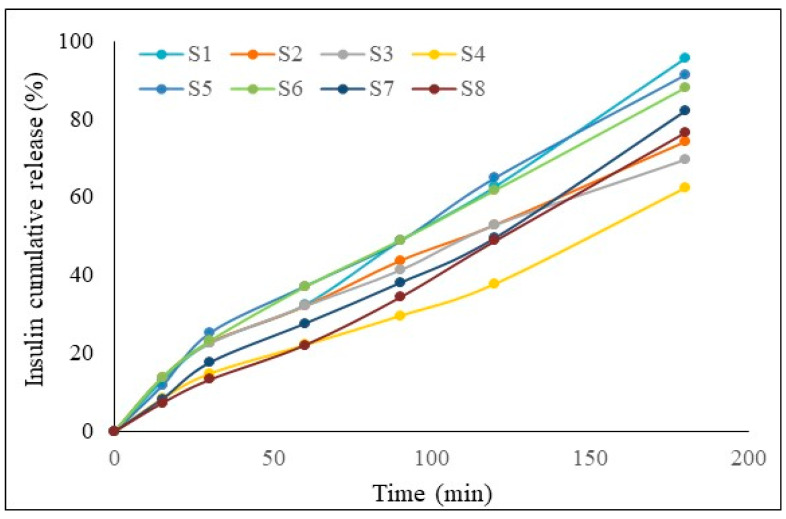
Cumulative release profiles of insulin from the hydrocolloidal systems.

In the first interval (Figure 3), all the samples had a good release, which is the most important, because the release should be quick in order to avoid a wash-out of the systems by mucociliary clearance.

### 2.4. Experimental Design Screening

The partial least squares (PLS) linear model was applied to evaluate the influence of the quantitative individual parameters (X1, X2, and X3) on the response parameters: work of adhesion (Y1), consistency index (Y2), and drug release (Y3). The statistical parameters that were used to evaluate the experiment were generated with Modde software, version 13; R2 was assessed as a statistical parameter to evaluate the goodness of fit for the experimental model and Q2 to estimate the goodness of prediction.

**Figure 4:**
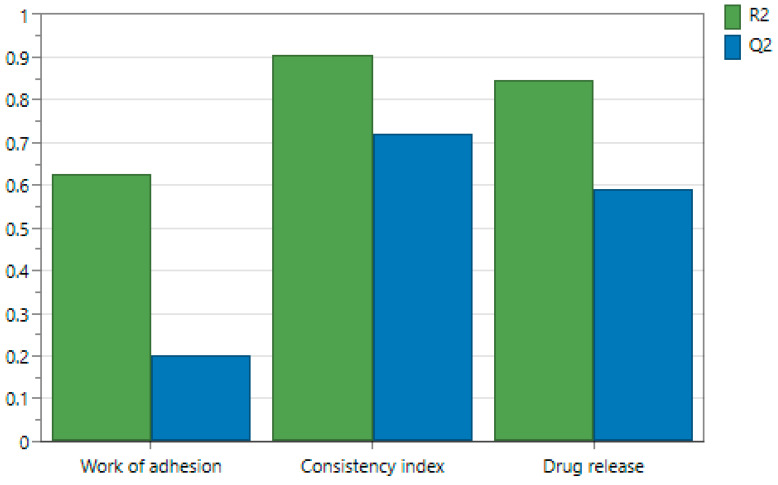
Graphical representation of statistical parameters R2 and Q2.

Taking into consideration that R2 was higher than 0.8 and Q2 > 0.5 for the response’s consistency index and drug release, and the response work of adhesion was R2 > 0.6 and Q2 > 0.2, the values fulfilled the expectations (Figure 4); thus, the model fits well and has predictive power.

Modde software was used to design the experimental plan and provide the 3D response surface and contour plots, which were used to observe the tendency of the response variables based on the changes in the formulation parameter.

**Figure 5:**
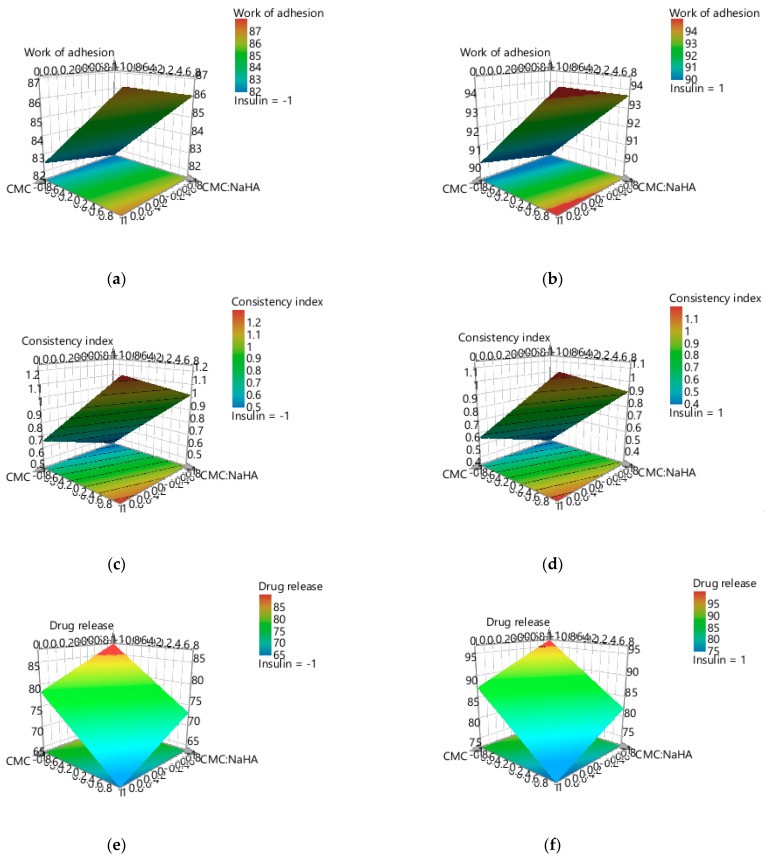
Three-dimensional surface plots representing the three responses when X3 is constant, (**a**) work of adhesion when X3 = −1, (**b**) work of adhesion when X3 = 1, (**c**) consistency index when X3 = −1, (**d**) consistency index when X3 = 1, (**e**) drug release when X3 = −1, (**f**) drug release when X3 = 1.

In Figure 5a–f, the tridimensional response surface plot is represented for each response parameter, varying X1—CMC concentration and X2—CMC/NaHA ratio, and maintaining the amount of insulin (X3) constant for the lower (−1) and upper (1) limits. At the lower amount of insulin, the work of adhesion varies proportionally with the concentration of CMC (Figure 5a), unlike the systems where the loading of insulin is at the upper level (Figure 5b). The consistency index is influenced proportionally by X1 and X2 in both situations (X3 = −1 and X3 = 1) (Figure 5c,d). On the other hand, the percentage of the drug release is influenced inversely proportionally by the concentration of CMC, and the ratio of the polymers impact negatively the release when the ratio is 1/2 for both amounts of insulin (Figure 5e,f).

**Figure 6:**
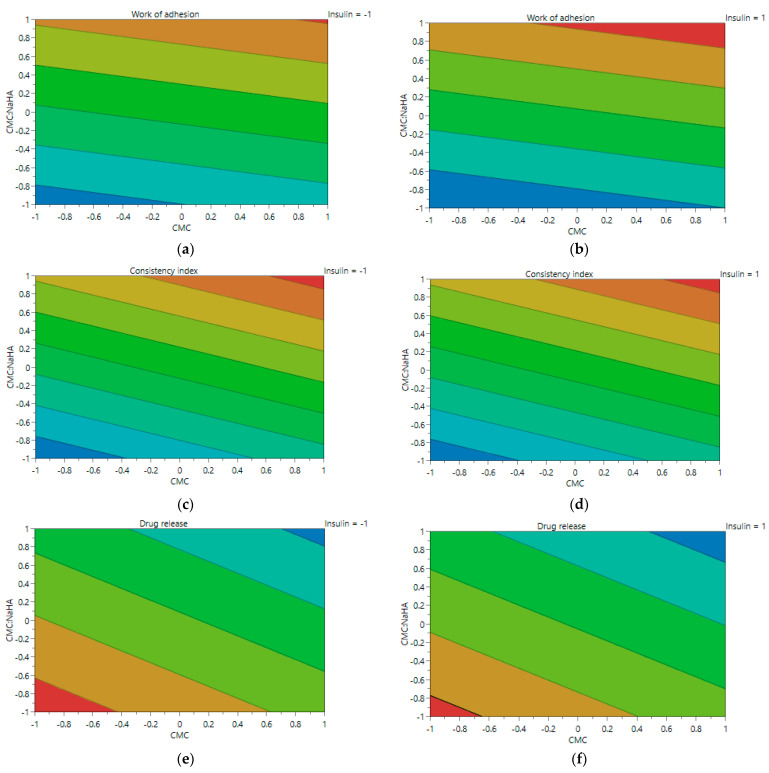
Contour plot for each response parameter, when X3 is constant, (**a**) work of adhesion when X3 = −1, (**b**) work of adhesion when X3 = 1, (**c**) consistency index when X3 = −1, (d) consistency index when X3 = 1, (**e**) drug release when X3 = −1, (**f**) drug release when X3 = 1.

The contour plot gives an overview of the experimental design, and based on Figure 6a–f, depicts the tendency of changing for each response parameter according to independent variables X1 and X2.

For all the responses analyzed, an ANOVA test was performed as a function of F and *p* values. The models were considered statistically significant when the *p* values were below 0.5.

Minitab statistical software was used to analyze the data and to complete the screening provided by the Modde program, by generating the regression equations and the Pareto charts for each response factor.

The work of adhesion (Y1) was negatively influenced by X2—the ratio between CMC and NaHA based on the regression equation (Equation (1)), where the *p* value is higher than 0.05 and the F value equals 2.25, although according to the statistical parameters, the model is not significant.

(1) Y1=74.8+0.96X1−9.27X2+0.753X3

**Figure 7:**
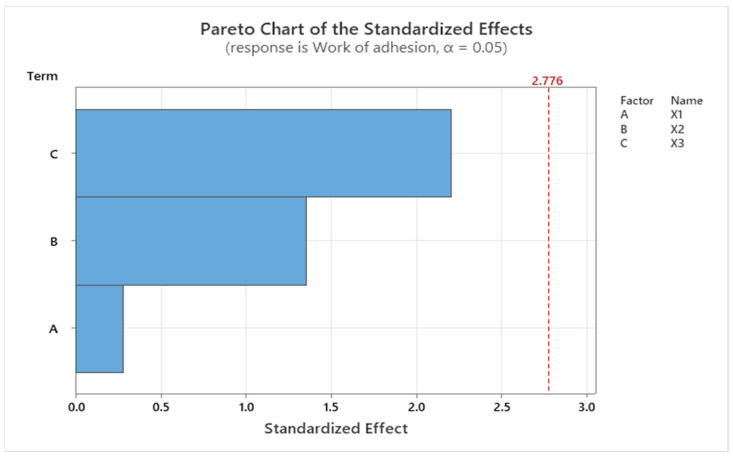
Pareto chart showing the standardized effect of the work of adhesion, where C (X3) is the amount of insulin, B (X2) is the CMC/NaHA ratio, and A (X1) is the CMC concentration.

Using a Pareto chart, the standardized effect for the response work of adhesion, explained previously by the linear regression equation, is presented in Figure 7. According to the graph, the main effect on the work of adhesion was given by the amount of insulin loaded in the systems.

Regarding the second response, the consistency index (Y2) regression model represented by Equation (2) was significant with a *p* value of 0.016 and F value of 12.76. As it was for Y1, the CMC/NaHA ratio had a negative impact on the consistency index response.

(2) Y2=1.576+0.226X1−1.177X2−0.0098X3

**Figure 8:**
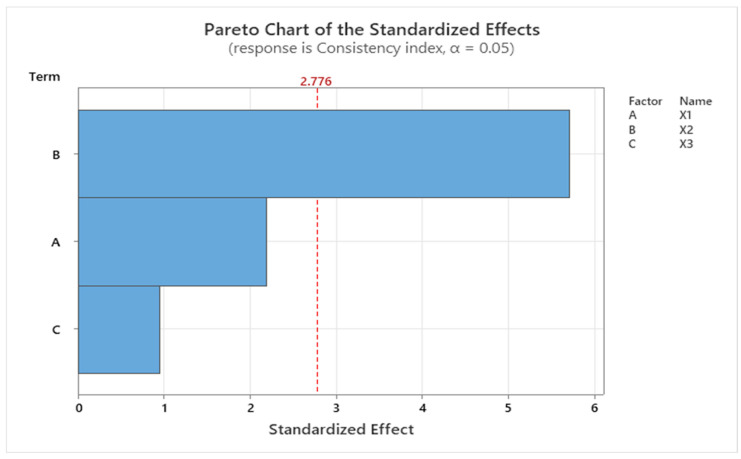
Pareto chart showing the standardized effect of the consistency index, where B (X2) is the CMC/NaHA ratio, A (X1) is the CMC concentration, and C (X3) is the amount of insulin.

Figure 8 represents the standardized effect for the consistency index using the Pareto chart, which shows the impact of the formulation variables on the response parameter. It confirms that the ratio between the two polymers had the main impact on the consistency index.

Following the mathematical modeling (Equation (3)), the drug-release response (Y3) was significantly influenced by X2—the CMC/NaHA ratio, and negatively influenced by X1—the concentration of CMC. The model analyzed was considered significant, with the *p* value = 0.041 and F value = 7.45.

(3) Y3=50.0−9.47X1+29.31X2+0.894X3

**Figure 9:**
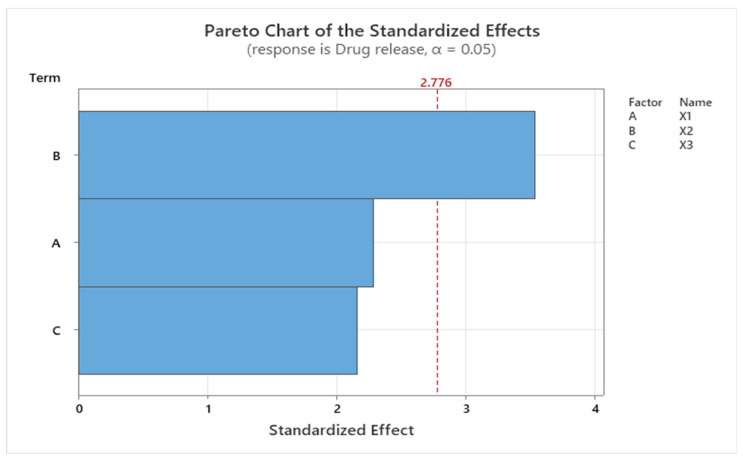
Pareto chart showing the standardized effect of drug release, where B (X2) is the CMC/NaHA ratio, A (X1) is the CMC concentration, and C (X3) is the amount of insulin.

The impact of the independent variable X2 (CMC/NaHA ratio) on the last response analyzed, Y3—drug release, is confirmed by the Pareto chart (Figure 9), where the standardized effect of the parameters is plotted.

**Figure 10:**
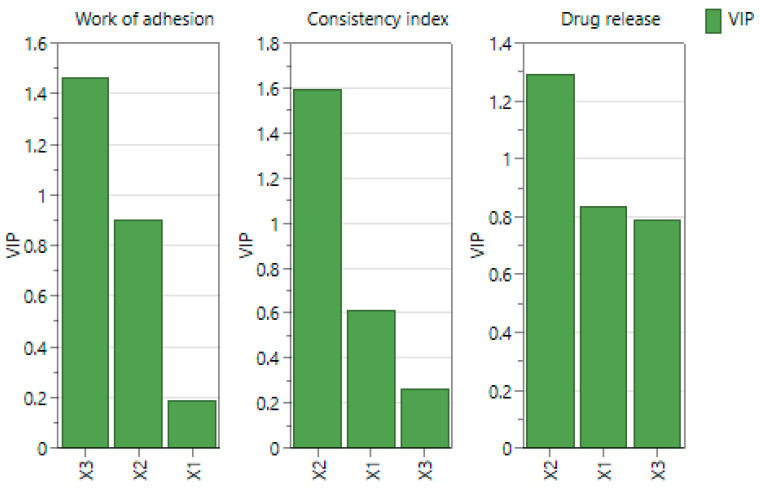
Variable importance in the projection (VIP) of the independent parameters, where X1 is the CMC concentration, X2 is the CMC/NaHA ratio, and X3 is the amount of insulin.

Combining all the interpretations previously mentioned, the importance of each parameter on each response variable can be seen. Based on the variable importance in the projection (Figure 10), it can be concluded that the work of adhesion was influenced by the amount of insulin (X3) and by the CMC/NaHA ratio (X2). For the consistency index response, the ratio between the polymers had a high significance. The CMC/NaHA ratio also had a pronounced meaning regarding the insulin drug release and the other two formulation parameters had similar significance. As noted before, the higher amount of NaHA influenced negatively the release of the active ingredient from the systems.

The use of Design of Experiments (DoE), as part of Quality by Design (QbD) principles, provides an overview of the experiment and performs a screening of the importance of the formulation parameters on the response parameters [54].

The predictive analysis was made with Modde and the optimal setpoint is presented in Table 5, with a probability of failure of 0.23%. Corroborating with the experimental results, samples S5, S7, and S8 scored close to the predicted system regarding the work of adhesion. The value for S7 fit the predicted value best regarding the consistency index. Systems S1, S5, S6, and S7 exceeded the cumulative release percentage compared to the optimal setpoint predicted. In conclusion, S7 is the most eloquent system when compared with the predictive setpoint.

The experimental plan was designed using Modde software, while Minitab software was employed to evaluate the relationship between the formulation variables and the response factors, highlighting the complementary strengths of both tools in the research process.

## 3. Discussion

The aim of this research was the formulation and the initial evaluation of hydrocolloidal systems containing insulin for intranasal administration, based on the intrinsic properties of CMC and NaHA. The in vitro study was based on the analysis of the influence of the formulation factors and the relationships between them on the work of adhesion and spreading at the level of the nasal mucosa, the flow behavior profiles, and on the cumulative quantity of the released drug.

The samples obtained were homogeneous, with a clear appearance and without impurities. The pH values of the hydrocolloid systems were at the tolerance limit of nasal mucosa [55]; pH lower than 4 would lead to nasal discomfort and damage to the nasal epithelium [56]. Both polymers, CMC and NaHA, are biocompatible with the mucosa and are not toxic [57].

The evaluation of the contact angle provides information about the ability of a liquid to wet a solid surface and its tendency to adhere to the solid. A contact angle lower than 90° indicates a high wetting capacity (wetting fluid) [58,59]. All the studied samples had recorded values of the contact angle lower than 90°, and thus we can state that the formulations obtained have the ability to wet the nasal mucosa.

The surface tension of liquid systems is an important parameter in the development of pharmaceutical products and can provide information about the quality of formulations, the bioavailability, and the absorption of substances [60,61]. The average surface tension of pharmaceutical preparations with intranasal administration is 30.3 mN/m to 44.9 mN/m [61,62]. The determinations made in this experiment showed that S1 had the closest value to the physiological surface tension of nasal mucosa, followed by S3, S6, and S7. However, all the samples had a higher surface tension compared with the average ST of the products with nasal administration.

The formulation of intranasal systems requires the use of tight junction modulators [1] and modulators of viscosity that have mucoadhesive properties [56]. As previously mentioned, both polymers have mucoadhesive properties; they have the ability to enhance the absorption of the active pharmaceutical ingredient through the tight junction of the epithelial mucosa.

Thus, the values obtained for the contact angle and the surface tension were used to calculate the work of adhesion and the work of cohesion, thereby determining the spreading capacity of the liquid formulations, based on the difference between the work of adhesion and the work of cohesion. All of these determinations referred to above are interconnected and help to evaluate the mucoadhesive properties of the hydrocolloidal systems based on adhesion and cohesion forces [63]. Mucoadhesion is defined as the intermolecular interactions between the formulations and the mucosa, quantified in this case by the work of adhesion. The work of cohesion indicates the interactions formed inside the systems, between the components of the product. Regarding the current study, both parameters recorded high values, but analysis from the point of view of the spreading coefficient, in the case of the series with a lower loading of insulin, S1 had a value of the spreading coefficient closer to 0, and for the series with an upper amount of insulin, S7 and S8. According to the literature, a liquid can be classified as spreadable when the contact angle is equal to 0° [64].

The same method was used by Spindler et al. to determine the spreading capacity of 10–30 mg/mL hyaluronate solution for nasal administration. The results indicated that contact angle values were below 90° and the surface tension was 46.82 mN m^−1^, with a high work of adhesion (over 80 mN m^−1^), and the spreading capacity was close to zero (−3.5 mN m^−1^ for the lowest concentration and −12.5 mN m^−1^ for the highest) [65].

Additionally, adhesion and cohesion forces can influence the flow behavior and viscosity of the formulations. The flow behavior of the eight systems was pseudoplastic, in accordance with the individual behavior of the polymers involved in the formulation of the hydrocolloidal systems and with other nasal products [66,67,68], and also with other formulations based on Carbopol, methyl cellulose, and glycerin, which were studied for the intranasal administration of insulin, according to the research of Ostrozka-Cieslik and collaborators [69].

Khodaverdi et al. also reported pseudoplastic behavior in casein-based hydrogel loaded with insulin, in which the viscosity of the systems showed a reduction when the shear rate was increased [70].

Viscosity is an important parameter that influences both the quality of the products and the bioavailability of the active pharmaceutical ingredient [52,71,72,73]. That is why systems with medium viscosity are preferred, which have an optimal contact time with the nasal mucosa but do not interfere with the physiological mucociliary clearance [74,75,76]. A high viscosity of solutions can lead to local adverse effects, such as nasal congestion or edema [77].

From the point of view of drug release, the expectation is that it should release rapidly to avoid the wash-out effect from mucociliary movements. The zero-order release kinetics has the advantage that the concentration of the drug is constant during a period of time [78]. All systems had an optimal release during the initial time interval; subsequently, S1 and S5 recorded the highest percentage of cumulative-released insulin, over 90%.

Mohamad et al. recorded a fast insulin release of 15–25% after 10 min and 65–90% after 30 min from hydroxypropyl methyl cellulose and polyvinyl alcohol films that contained insulin for nasal administration [79].

The data reported by Nazar and collaborators describe the in vitro release of insulin from a thermo-responsive gel based on N-trimethyl chitosan chloride, where a burst release was recorded during the first 12 min, with circa 70% insulin release [80].

The study conducted by Muntu et al. showed rapid release of insulin from a nasal powder, using trehalose as a stabilizer, in the first 30 min (49–60%) and a cumulative release of 79–88% after 1 h [81].

According to de Von Zuben et al., who investigated the controlled release of insulin from the liposomes after intranasal administration, in the first 2 h there was immediate release of the insulin, and a rapid effect was obtained [82]. The same researchers reported that the same burst effect was obtained in the first 2 h, based on their study of the comparative release of insulin from insulin solution, hydroxyethylcellulose-based hydrogels with insulin, or HEC-based hydrogel containing insulin-loaded liposomes after nasal administration [68].

In another study, Sharma and collaborators reported that nanoparticles based on chitosan and loaded with insulin for intranasal administration had a cumulative drug release of 93.32%, with a significant burst effect of 22.25% during approximately the first 30 min [83].

Regarding the research performed by other authors, Li et al. described a rapid release of insulin in the first 1 h from deep eutectic solvents for nasal delivery, which combined malic acid and choline chloride, and a total release of 75% after 5 h of experiments [84].

Using the 3D-response surface-plot representation and the contour plot for each response parameter generated by Modde software, combined with the regression equations and Pareto charts generated by Minitab software, the results help show the impact of each parameter on the hydrocolloidal systems. The screening of the experimental design, especially in the initial assessment, provides valuable information regarding the significance of each formulation’s factors, the reproducibility, and the validity of the model, and can predict the future experiments. Corroborating all the results obtained with the predicted analysis, the systems with 1% chitosan and CMC/NaHA 1/1 ratio were the most effective, regardless the loading of insulin. Even if the percentage of cumulative release of the active ingredient was high for S1 and S5, the system S7 fit better in the predictive analysis and can be included in future research.

Further studies can be carried out on the hydrocolloidal liquid formulations regarding the administration devices that have advanced from sprays, nebulizers, nasal pumps, or single-dose vials, to innovative systems with precise release at the level of the olfactory region in the nasal cavity (precision olfactory delivery devices) [1,5,55,77,85].

## 4. Materials and Methods

### 4.1. Materials

Carboxymethyl chitosan with viscosity 200–300 cPs (1% water) and Sodium Hyaluronate purchased from Sigma Aldrich (Saint Louis, MO, USA). Human insulin (Humulin R 100 UI/mL, Lilly France, Fegersheim, France) was bought from a local pharmacy. Ultrapure distilled water was provided by a Milli-Q water purification system (Merck Millipore, Bedford, MA, USA) and was used as solvent for the preparation of the formulations.

### 4.2. Preparation of the Hydrocolloidal Systems and Experimental Design

CMC was dissolved in ultrapure distilled water under continuous stirring at room temperature. Solutions of CMC 1% *w*/*v* and 2% *w*/*v* were obtained. The solution of NaHA 1% w/v was obtained by dissolving the NaHA in ultrapure water. After overnight storage of all the solutions in a refrigerator, the final samples were mixed according to the appropriate ratio, and then the insulin solution was added under continuous homogenization. A 2^3^ fractional factorial plan was developed, in which there are three independent variables at two levels of variation: X1—concentration of the CMC, X2—the ratio between CMC and NaHA, and X3—the amount of insulin. The design of the experiment is presented in Table 6.

The objective of this research was to develop and characterize the hydrocolloidal systems based on CMC and NaHA for nasal administration of insulin.

### 4.3. Physico-Chemical Characterization of Hydrocolloidal Systems

#### 4.3.1. Visual Aspect and pH Determination

The formulations obtained were visually examined to observe the aspect, color, and instability (precipitation, separation or other changes), if any, that may have occurred during the preparation.

The pH was determined using a Mettler-Toledo pH meter (Mettler-Toledo GmbH, Im Langacher 44, 8606 Greifensee, Switzerland) at room temperature. Before the determinations, the pH meter was calibrated with buffer solutions at pH 4 and 7. The accuracy of the measurement was ±0.01 pH.

#### 4.3.2. Contact Angle Determination

The contact angle measurements were performed with CAM 101 (KSV Instruments Ltd., Espoo, Finland) equipped with a Hamilton syringe, at room temperature. The sessile drop method was used. The drops at the contact with the solid surface were analyzed using a digital video camera. Based on the images, the CA was automatically calculated using the Young–Laplace method with the software supplied by the producer of the goniometer.

#### 4.3.3. Surface Tension Determination

The surface tension measurement was made following the same technique as presented for CA, but the pedant drop method was used for this parameter. The ST was calculated based on the images of the drops that were captured just before detachment from the needle.

#### 4.3.4. Work of Adhesion, Work of Cohesion, and Spreading Coefficient Determinations Based on Superficial Properties

The adhesion properties of the systems were evaluated using the work of adhesion, which was calculated based on the CA and ST values using the Young–Dupré Equation (4):

(4) Wa=ST(1+cosCA)

where Wa is work of adhesion (mN/m), ST is surface tension (mN/m), and CA is contact angle (rad).

The work of cohesion represents the intermolecular bonding between the formulation parameters and can influence the spreading capacity and the viscosity of the systems. It is determined based on Equation (5):

(5) Wc=2ST

where Wc is work of cohesion (mN/m) and ST is surface tension (mN/m).

The system display of on the nasal mucosa was assessed using the spreading coefficient. Equation (6) was used to obtain the spreading coefficient.

(6) S=Wa–Wc

where S is spreading coefficient (mN/m), Wa is work of adhesion (mN/m), and Wc is work of cohesion (mN/m).

#### 4.3.5. Rheology Analysis

The rheological analysis was performed at 35 ± 0.5 °C with a Lamy RM100 rheometer (Lamy Rheology Instruments, Champagne au Mont d’Or, France) equipped with CP2000 Plus thermostat. A cone-plate CP6020 (2°, 60 mm diameter) was used, and the rotational speed applied was between 0.3 rpm and 60 rpm. The mathematical power law model was applied (Equation (7)), which shows the relation between the shear stress and shear rate, in order to analyze the flow behavior:

(7) $$\tau =K\cdot {\dot{\gamma }}^{n}$$

where τ is shear stress (Pa), K is consistency index (Pa·s^n^), $\dot{\gamma }$ is shear rate (s^−1^), and n is flow index.

### 4.4. In Vitro Drug Release

In vitro release rates were evaluated using a Franz-diffusion cell system (Teledyne Hanson Research, Hanson, MA, USA). The experiment was performed at 35 ± 0.5 °C in phosphate-buffered saline (PBS) at pH 7.4. PBS was added in the receptor compartments (7 mL) and stirred with magnetic bars at 300 rpm. The receptor and donor compartments were separated by cellulose acetate membrane diffusion (area 1.77 cm^2^). The donor phase contained 20 IU/mL or 30 IU/mL insulin. At predefined times during a period of 2 h, samples were taken from the receptor compartments and were replaced with fresh PBS. The amount of insulin diffused was determined spectrophotometrically at 271 nm. The insulin-release kinetics followed the Higuchi model (Equation (8)), zero-order model (Equation (9)) and power law model (Equation (10)) [72,86], and the cumulative percentage was plotted as a function of time.

Higuchi model:

(8) $${\frac{m_{t}}{m}}_{\infty }=k\cdot t^{0.5}$$

Zero-order model:

(9) $${\frac{m_{t}}{m}}_{\infty }=k\cdot t$$

Power law model:

(10) $${\frac{m_{t}}{m}}_{\infty }=k\cdot t^{n}$$

where ${\frac{m_{t}}{m}}_{\infty }$ is the fraction of drug released at time *t*, *k* is the kinetic constant, and *n* is the release exponent.

### 4.5. Experimental Design Screening

The influence of the experimental factors on the response parameters was evaluated using Modde statistical software (Version 13.1, Sartorius, Goettingen, Germany) [87]. The partial least squares (PLS) linear method was used to fit the experimental data. The responses were defined as work of adhesion (Y1), consistency index (Y2), and cumulative drug release (Y3). An ANOVA statistical evaluation was performed. A linear regression equation was generated for each response parameter with Minitab software [88] to analyze the influence of the independent factors (X1, X2, and X3) and to generate the Pareto charts, which show the impact of the independent variable on the responses [89].

## 5. Conclusions

The presented research offers preliminary information regarding the potential use of hydrocolloidal systems in the nasal administration targeting nose-to-brain delivery. The two polymers, CMC and NaHA, were chosen based on their mucoadhesive characteristics, which are sustained by their biocompatibility, biodegradability, and non-toxic properties.

The pH was in the physiological tolerability limit for all eight systems. The wetting capacity of the formulations was confirmed by the CA evaluation, where the values for all the systems were below 90°. The ST values were near the value of the nasal mucosa, especially for S1. The mucoadhesion properties were confirmed by the work of adhesion results for all the formulations. The pseudoplastic behavior obtained for the hydrocolloidal systems is adequate for the products with nasal administration.

The initial assessment also included the in vitro cumulative release of insulin, where S1 and S5 exhibited a high percentage of cumulative release and all the systems had a rapid release of the pharmaceutical active ingredient, which was according to our expectations, but further investigations should be conducted.

According to the results from the two programs used in this research, for the two responses, consistency index and drug release, where the model had statistical significance, the ratio between CMC and NaHA had the main impact on the response variables, and was followed by the concentration of CMC.

The topic addressed in this study, insulin administered intranasally for diseases associated with the central nervous system, is still very broad. Additional studies are still needed to investigate its effects and transport from the nose to the brain. The formulations in this research were preliminarily analyzed in vitro from the point of view of the influence of the formulation factors on their superficial, rheological, and release properties. In order to evaluate the permeation, bioavailability, and toxicology on the systems, these formulations are suitable for further animal and human research.

## Figures and Tables

**Table 1:** Table of formulations and results for pH, contact angle, and surface tension.

System	X1 CMC (% *w*/*v*)	X2 CMC/NaHA Ratio	X3 Insulin (IU/mL)	pH	Contact Angle (°)	Surface Tension (mN/m)
S1	1	1/1	20	6.65	66.35 ± 0.64	57.19 ± 0.24
S2	2	1/1	20	6.57	65.48 ± 2.06	60.51 ± 0.05
S3	1	1/2	20	6.68	68.79 ± 1.56	58.85 ± 0.77
S4	2	1/2	20	6.64	64.68 ± 0.73	62.07 ± 0.71
S5	1	1/1	30	6.68	61.73 ± 0.75	60.91 ± 0.49
S6	2	1/1	30	6.62	64.10 ± 0.65	59.01 ± 0.32
S7	1	1/2	30	6.72	50.47 ± 0.26	59.65 ± 0.72
S8	2	1/2	30	6.65	58.43 ± 0.52	62.46 ± 0.30

where CMC is carboxymethyl chitosan and NaHA is sodium hyaluronate.

**Table 2:** Work of adhesion, work of cohesion and spreading coefficient results.

System	Wa	Wc	S
S1	82.84 ± 0.27	114.38 ± 0.48	−31.54 ± 0.7
S2	85.62 ± 1.95	121.02 ± 0.1	−35.40 ± 2.02
S3	80.14 ± 2.75	117.7 ± 2.72	−37.56 ± 1.45
S4	88.62 ± 1.16	124.14 ± 1.43	−35.52 ± 0.88
S5	89.76 ± 0.26	121.83 ± 0.98	−32.06 ± 0.96
S6	84.79 ± 0.8	118.03 ± 1.31	−33.24 ± 0.57
S7	97.62 ± 1.1	119.3 ± 1.44	−21.68 ± 0.23
S8	95.17 ± 0.45	124.93 ± 0.23	−29.76 ± 0.21

where Wa is the work of adhesion (mN/m); Wc is the work of cohesion (mN/m), and S is the spreading coefficient (mN/m).

**Table 3:** Power law model parameters for all eight systems.

Sample	S1	S2	S3	S4	S5	S6	S7	S8
**K**	0.261	0.841	1.054	1.187	0.393	0.475	0.897	1.095
**n**	0.611	0.539	0.526	0.549	0.670	0.620	0.576	0.546
**R^2^**	0.9980	0.9926	0.9960	0.9952	0.9969	0.9953	0.9958	0.9954

where K is the consistency index (Pa·s^n^); n is the flow index, and R^2^ is the determination coefficient.

**Table 4:** Comparison of determination coefficients (R) for the Higuchi, zero-order, and power law kinetic models; kinetic parameters specific to the zero-order and power law models; and the cumulative drug-release percentage.

System	Determination Coefficient (R^2^)			Kinetic Parameters			Drug Released (%)
	Higuchi Model	Zero-Order Model	Power Law Model	Zero-Order Model	Power Law Model		
				Kinetic Constant (1/min)	Kinetic Constant (1/min^n^)	Release Exponent (n)	
1	0.9248	0.9959	0.9934	0.538	0.891	0.896	95.91
2	0.9710	0.9819	0.9962	0.445	1.885	0.703	74.35
3	0.9790	0.9771	0.9972	0.427	2.146	0.668	69.86
4	0.9128	0.9934	0.9852	0.342	0.522	0.913	62.53
5	0.9515	0.9913	0.9958	0.534	1.419	0.799	91.53
6	0.9585	0.9899	0.9980	0.517	1.554	0.774	88.06
7	0.9026	0.9962	0.9905	0.444	0.482	0.983	82.18
8	0.8857	0.9978	0.9968	0.412	0.282	1.076	76.65

**Table 5:** Optimal setpoint generated by Modde software.

Response	Optimal Setpoint
Work of adhesion (mN/m)	92.55
Consistency index (Pa·s^n^)	0.89
Drug release (%)	81.64

**Table 6:** Experimental design.

Factors	Independent Variables	Levels of Variation	
		Lower (−1)	Upper (+1)
X1	Carboxymethyl chitosan (% *w*/*v*)	1	2
X2	CMC/NaHA ratio	1/1	1/2
X3	Insulin (IU/mL)	20	30

## Data Availability

The data presented in this study are available in the article.
